# Biotyping of Multidrug-Resistant *Klebsiella pneumoniae* Clinical Isolates from France and Algeria Using MALDI-TOF MS

**DOI:** 10.1371/journal.pone.0061428

**Published:** 2013-04-19

**Authors:** Meryem Berrazeg, Seydina M. Diene, Mourad Drissi, Marie Kempf, Hervé Richet, Luce Landraud, Jean-Marc Rolain

**Affiliations:** 1 Aix-Marseille Université, Unité de Recherche en Maladies Infectieuses et Tropicales Emergentes (URMITE), UM63, CNRS 7278, IRD 198, Inserm 1095, IHU Méditerranée Infection, Faculté de Médecine et de Pharmacie, Marseille, France; 2 Laboratoire Antibiotiques, Antifongiques: Physico-Chimie, Synthèse et Activité Biologiques, Faculté des Sciences de la Nature, de la Vie, de la Terre et de l’Univers, Université Abou Bekr Belkaid, Tlemcen, Algérie; 3 Laboratoire de Bactériologie, Institut de Biologie en santé – PBH, CHU, Angers, France; 4 Laboratoire de Bactériologie - Hôpital l’Archet 2, CHU de Nice, Nice, France; Rockefeller University, United States of America

## Abstract

**Background:**

*Klebsiella pneumoniae* is one of the most important pathogens responsible for nosocomial outbreaks worldwide. Epidemiological analyses are useful in determining the extent of an outbreak and in elucidating the sources and the spread of infections. The aim of this study was to investigate the epidemiological spread of *K. pneumoniae* strains using a MALDI-TOF MS approach.

**Methods:**

Five hundred and thirty-five strains of *K. pneumoniae* were collected between January 2008 and March 2011 from hospitals in France and Algeria and were identified using MALDI-TOF. Antibiotic resistance patterns were investigated. Clinical and epidemiological data were recorded in an Excel file, including clustering obtained from the MSP dendrogram, and were analyzed using PASW Statistics software.

**Results:**

Antibiotic susceptibility and phenotypic tests of the 535 isolates showed the presence of six resistance profiles distributed unequally between the two countries. The MSP dendrogram revealed five distinct clusters according to an arbitrary cut-off at the distance level of 500. Data mining analysis of the five clusters showed that *K. pneumoniae* strains isolated in Algerian hospitals were significantly associated with respiratory infections and the ESBL phenotype, whereas those from French hospitals were significantly associated with urinary tract infections and the wild-type phenotype.

**Conclusions:**

MALDI-TOF was found to be a promising tool to identify and differentiate between *K. pneumoniae* strains according to their phenotypic properties and their epidemiological distribution. This is the first time that MALDI-TOF has been used as a rapid tool for typing *K. pneumoniae* clinical isolates.

## Introduction

*Klebsiella pneumoniae* are ubiquitous in nature and have two common habitats; one is the environment, including surface water, sewage, soils and plants [1], and the other is mammalian mucosal surfaces [2]. In humans, *K. pneumoniae* can be present in the intestinal tract, nasopharynx, and on the skin [3]. It is one of the most common Gram-negative bacteria encountered by clinicians worldwide as a cause of infections in humans [4] and is responsible for outbreaks due to the propagation of different clones associated with opportunistic infections in individuals with impaired immune defenses, such as diabetics, alcoholics and hospitalized patients with indwelling devices [3]. In the hospital environment, the principal reservoirs for *K. pneumoniae* transmission are blood products, contaminated medical equipment, the gastrointestinal and respiratory tracts of patients and the hands of hospital personnel [5]. The hospital-acquired infections caused by this organism mainly include pneumonia, septicemia, urinary tract infections and soft tissue infections [6].

Increased *K. pneumoniae* infections are also associated with an increase in multidrug-resistant (MDR) strains, especially those producing extended-spectrum beta-lactamases (ESBLs) [7] associated with the prior use of antibiotics, particularly the cephalosporins [8]. Furthermore, several carbapenemase-encoding genes have been described in *K. pneumoniae* species, including class A beta-lactamase KPC, class B beta-lactamases NDM, IMP and VIM, and class D beta-lactamase OXA-48 [9]. The hospital epidemiology of these infections is often complex because multiple clonal strains causing focal outbreaks may co-exist with sporadic strains that also have a reservoir in the community [10]. Infections caused by multidrug-resistant *K. pneumoniae* strains have been associated with adverse clinical outcomes, including increased mortality, prolonged hospital stays and increased economic costs [11].

Therefore, epidemiological typing is useful in determining the extent of an outbreak and in investigating the sources, the reservoir and the spread of bacterial infections. Various methods, including protein profiling by sodium dodecyl sulfate polyacrylamide gel electrophoresis (SDS-PAGE) and DNA profiling by multilocus sequence typing (MLST), restriction fragment length polymorphism (RFLP) and pulsed field gel electrophoresis (PFGE), have been used for the epidemiological typing of *K. pneumoniae* isolates [12]–[14]. However, most of these methodologies are time consuming, laborious, require special skills and are unsuitable for use in routine clinical laboratories [15], [16]. In recent years, several reports have shown the feasibility of using matrix-assisted laser desorption ionization (MALDI)-time of flight (TOF) mass spectrometry (MS) to rapidly identify microorganisms [17]. There are only a few studies that have evaluated this method as a rapid tool to classify bacterial species at the strain level [18], [19]. However, there are some recent examples of the use of MALDI-TOF MS for the rapid identification and typing of a limited number of clinical strains, such as *Streptococcus pyogenes* [20] or *Klebsiella pneumoniae* [19].

Here, we report the evaluation of MALDI-TOF MS as a rapid and powerful tool for determining the epidemiological distribution of a large series of *K. pneumoniae* clinical strains of different origins from patients with various infectious syndromes and the correlation between the pathotypes, geographic locations and clonalities of these strains using MALDI-TOF MS and data-mining approaches.

## Results

### Clinical Data

The mean age of the infected patients was 53 years and was similar when comparing patients hospitalized in Algerian hospitals (53 years: range 12 days to 84 years) and those from French hospitals (53.1 years: range 1 day to 97 years). The male/female ratio was 1/1. Among the 535 isolates, 172 (32.1%) were retrieved from intensive care units (ICUs), 160 (29.9%) from infectious diseases wards, 40 (7.5%) from surgical wards, 38 (7.1%) from emergency wards, 22 (4.1%) from pediatric wards, 21 (3.9%) from trauma wards, 21 (3.9%) from neurology wards, 16 (3.0%) from internal medicine wards, 14 (2.6%) from nephrology wards, 14 (2.6%) from gastroenterology wards, and 17 (3.2%) from gynecology, cardiology, or geriatric medicine wards. *K. pneumoniae* strains were isolated from various clinical samples that originated as follows: 189 (35.3%) from urine, 113 (21.1%) from tracheal aspirate, 73 (13.6%) from pus, 67 (12.5%) from blood culture, 36 (6.7%) from rectal swab and 57 (10.6%) from other different clinical specimens.

### Bacterial Identification

Using MALDI-TOF MS, all *K. pneumoniae* strains (100%) were identified with score values >1.9 using the Bruker Biotyper software.

### Antibacterial Susceptibility Testing of *Klebsiella pneumoniae* Isolates

As shown in Table 1, the overall resistances (i.e., resistant+intermediate percentage (R+I%)) to various antibiotics were as follows: ampicillin, amoxicillin and ticarcillin (100%), amoxicillin/clavulanic acid (55.5%), cefalotin (53.8%), cefoxitin (7.7%), ceftazidime (51.2%), cefotaxime and ceftriaxone (51.0%), gentamicin (48.2%), tobramycin (72.4%), amikacin (26.4%), ciprofloxacin (45.1%), and trimethoprim/sulfamethoxazole (45.7%). All isolates were sensitive to imipenem and colistin. The *K. pneumoniae* strains from Algeria had significantly higher percentages of resistance to antibiotics compared to those from France (*p*<0.0001). The prevalence of resistance to third generation cephalosporin in Algeria was 88.7% compared with France (26.5%).

**Table 1:** Origin and repartition of strains used in this study.

Hospital		Department		Sex/Source		Age	Period study	Sample origin	
Annaba	18	Intensive care unit	18	FemaleMale	810	22 to 62 years24 to 79 years	From March 2009to October 2010	Tracheal aspirateUrineBlood culture	1242
Sidi Bel Abbes	28	Intensive care unitSurgeryInternal medicineNephrologyTraumaEmergency	1472311	FemaleMaleEnvironment	15112	11 to 66 years7 to 75 years	From October 2009to February 2011	Tracheal aspirateUrineEnvironmentBedsorePusProfound swab	9322111
Tlemcen	72	Intensive care unitSurgeryGynecologyNeurologyPediatricsTrauma	48512610	FemaleMaleEnvironment	214011	24 to 70 years7 to 74 years	From August 2008to January 2011	Tracheal aspirateUrineEnvironmentPusRectal swabProfound swab	2831111181
Oran	93	Intensive care unitSurgeryNeurologyPediatricsTrauma	6081096	FemaleMaleEnvironment	36525	1 day to 69 years4 to 73 years	From April 2008to March 2011	Tracheal aspirateUrineEnvironmentPusRectal swabVaginal swab	521351391
Angers	100	Infectious diseases	100	FemaleMale	5644	22 to 97 years1 day to 97 years	From January 2008to March 2011	Tracheal aspirateCatheterUrineBlood culturePusRectal swabVaginal swab	335818396
Marseille	170	CardiologySurgeryGastroenterologyGeriatric medicineGynecologyInfectious diseasesInternal medicineNephrologyNeurologyPediatricsIntensive care unitTraumaEmergency	7201436614119732437	FemaleMale	9476	6 month to 94 years1 day to 86 years	From January 2009to Jun 2009	Tracheal aspirateCatheterUrineBedsoreBlood culturePusProfound swabSubcutaneous swabND	9110812166117
Nice	54	Infectious diseases	54	FemaleMale	21 33	1 day to 88 years1 day to 88 years	From January 2010to October 2010	Blood culturePus	2628

Origin and repartition of 535 *Klebsiella pneumoniae* strains isolated between January 2008 and March 2011.

Six resistance phenotypes were found based on susceptibility to beta-lactams (Table 2). These comprised 240 (44.8%) wild-type, 7 (1.3%) inhibitor-resistant TEM penicillinase, 11 (2.0%) high-level penicillinase, 3 (0.6%) cephalosporinase, 240 (44.8%) extended-spectrum beta-lactamase (ESBL), and 34 (6.3%) ESBL associated with cephalosporinase. The difference in antibiotic resistance rate was significant between Algerian and French strains (*p*<0.0001). Overall, the percentage of non-ESBL-producing strains was higher in France (73.1%) than in Algeria (11.3%), while that of ESBL-producing strains was higher in Algeria (88.6%) compared with France (26.8%).

**Table 2:** Antibiotic susceptibility testing results of *Klebsiella pneumoniae* strains.

	*K. pneumoniae* strains of Algeria (n = 211)						*K. pneumoniae* strains of France (n = 324)						*p* values*	Total of *K. pneumoniae* strains (n = 535)					
	S	%	I	%	R	%	S	%	I	%	R	%		S	%	I	%	R	%
**AM**	0	0,0	0	0,0	211	100,0	0	0,0	0	0,0	324	100,0	–	0	0,0	0	0,0	535	100,0
**AMX**	0	0,0	0	0,0	211	100,0	0	0,0	0	0,0	324	100,0	–	0	0,0	0	0,0	535	100,0
**AMC**	27	12,8	75	35,5	109	51,6	196	67,6	40	13,8	54	18,6	<0.0001	223	44,5	115	22,9	163	32,5
**TIC**	0	0,0	0	0,0	211	100,0	0	0,0	0	0,0	324	100,0	–	0	0,0	0	0,0	535	100,0
**CF**	19	9,0	0	0,0	192	91,0	228	70,4	2	0,6	94	29,0	<0.0001	247	46,2	2	0,4	286	53,4
**FOX**	182	86,2	25	11,8	4	1,9	259	97,0	3	1,1	5	1,9	–	441	92,2	28	5,8	9	1,9
**CTX**	24	11,4	0	0,0	187	88,6	238	73,4	4	1,2	82	25,3	<0.0001	262	49,0	4	0,7	269	50,3
**CAZ**	23	10,9	12	5,7	176	83,4	238	73,4	50	15,4	36	11,1	<0.0001	261	48,8	62	11,6	212	39,6
**CRO**	24	11,4	0	0,0	187	88,6	238	73,4	4	1,2	82	25,3	<0.0001	262	49,0	4	0,7	269	50,3
**IMP**	211	100,0	0	0,0	0	0,0	324	100,0	0	0,0	0	0,0	–	535	100,0	0	0,0	0	0,0
**AN**	143	67,8	8	3,8	60	28,4	199	78,3	44	17,3	11	4,3	<0.0001	342	73,5	52	11,2	71	15,3
**GN**	26	12,3	3	1,4	182	86,2	243	78,9	1	0,3	64	20,8	<0.0001	269	51,8	4	0,8	246	47,4
**TM**	21	9,9	15	7,1	175	82,9	62	68,9	0	0,0	28	31,1	<0.0001	83	27,6	15	5,0	203	67,4
**CIP**	60	28,4	8	3,8	143	67,8	224	72,7	2	0,6	81	26,4	<0.0001	284	54,8	10	1,9	224	43,2
**CS**	211	100,0	0	0,0	0	0,0	324	100,0	0	0,0	0	0,0	–	535	100,0	0	0,0	0	0,0
**SXT**	57	27,0	7	3,3	147	69,7	223	73,1	2	0,6	80	26,2	<0.0001	280	54,3	9	1,7	227	44,0

* the *p* values compare the percentage of resistance and sensitivity between Algerian and French strains,

**S**: Sensitive, **I** : Intermediate, **R** : Resistant, **AM**: Ampicillin, **AMX**: Amoxicillin, **AMC**: Amoxicillin/clavulanic acid, **TIC**: Ticarcillin, **CF**: Cefalotin, **FOX**: Cefoxitin, **CAZ**: Ceftazidime, **CTX**: Cefotaxime, **CRO**: Ceftriaxone, **IMP**: Imipenem, **GN**: Gentamicin, **AN**: Amikacin, **TM**: Tobramycin, **CIP**: Ciprofloxacin, **SXT**: Trimethoprim/Sulfamethoxazole, **CS**: Colistin.

### Data Mining Analysis of *Klebsiella pneumoniae* MSP Dendrogram

**Figure 1:**
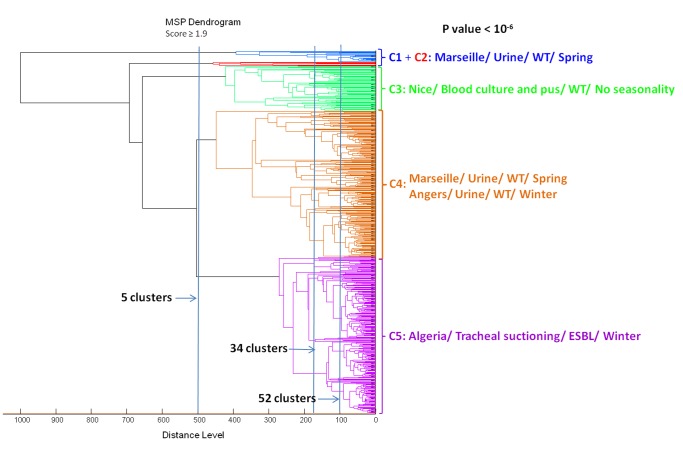
Geographic location of hospitals implicated in this study. 1: Tlemcen, 2: Sidi Bel Abbes, 3: Oran, 4: Annaba, 5: Marseille, 6: Nice, 7: Angers.

The MSP dendrogram revealed five distinct clusters according to an arbitrary cut-off at the distance level of 500 (Figure 1). Data mining analysis of the five clusters using PASW 17.0 software showed that *K. pneumoniae* strains isolated in Algerian hospitals (Tlemcen, Sidi Bel Abbes, Oran, Annaba) were significantly associated with respiratory tract infections and ESBL phenotype in the fifth cluster (*p*<0.0001), whereas *K. pneumoniae* strains isolated in Marseille hospitals were significantly associated with urinary tract infections and wild type phenotype in the first, the second and the fourth clusters (*p*<0.0001). *K. pneumoniae* strains isolated in Angers hospitals were associated with urinary tract infections and wild type phenotype in the fourth cluster (*p*<0.0001). Conversely, *K. pneumoniae* strains isolated in Nice hospital were associated with blood cultures and pus samples and wild type phenotype in the third cluster (*p*<0.0001) (Table 3). All the details of the distribution of *K. pneumoniae* strains into the five clusters according to the dendrogram are given in supplementary Table S1. Interestingly, clustering the strains according to the arbitrary distance levels of 180 and 100 significantly clustered strains from the same hospital into the same cluster. At the distance level of 180, 34 distinct clusters were identified, and at the distance level of 100, 52 distinct clusters were identified (Table S2). For example, at the distance level of 100, one cluster containing 15 strains showed that all of them were from Marseille hospital, a second cluster contained three strains (all from Nice hospital), and a third cluster contained ten strains (eight out of ten were from Angers hospital (*p*<0.0001)).

**Table 3:** Antibacterial resistance phenotypes of *Klebsiella pneumoniae* strains.

	Tlemcen(%)	Sidi BelAbbes (%)	Oran(%)	Annaba(%)	TotalAlgeria (%)	Angers(%)	Nice(%)	Marseille(%)	TotalFrance (%)	Total(%)
**ESBL**	58 (80,5)	21 (75,0)	62 (66,7)	18 (100,0)	159 (75,3)	24 (24,0)	7 (13,0)	50 (29,4)	81 (25,0)	240 (44,8)
**ESBL+Case**	9 (12,5)	3 (10,7)	16 (17,2)	0 (0,0)	28 (13,3)	6 (6,0)	0 (0,0)	0 (0,0)	6 (1,8)	34 (6,3)
**Case**	1 (1,4)	0 (0,0)	0 (0,0)	0 (0,0)	1 (0,5)	2 (2,0)	0 (0,0)	0 (0,0)	2 (0,6)	3 (0,6)
**Pase High Level**	1 (1,4)	1 (3,6)	2 (2,1)	0 (0,0)	4 (1,9)	0 (0,0)	7 (13,0)	0 (0,0)	7 (2,2)	11 (2,0)
**Pase IRT**	0 (0,0)	2 (7,1)	0 (0,0)	0 (0,0)	2 (0,9)	2 (2,0)	1 (1,8)	2 (1,2)	5 (1,5)	7 (1,3)
**Wild Type**	3 (4.2)	1 (3,6)	13 (14,0)	0 (0,0)	17 (8,0)	66 (66,0)	39 (72,2)	118 (69,4)	223 (68,8)	240 (44,8)
**Total**	72	28	93	18	211	100	54	170	324	535

ESBL: Extended-spectrum beta-lactamase, Case: Cephalosporinase, ESBL+Case: Extended-spectrum beta-lactamase associated to Cephalosporinase phenotype, Pase: Penicillinase, Pase IRT: inhibitor-resistant TEM penicillinase.

The monthly distribution of *K. pneumoniae* strains for the January 2008 - March 2011 period was determined by PASW 17.0 software according to clusters. Strains from Angers and Algerian hospitals were significantly correlated with winter (January-March) (*p*<0.0001), whereas, strains from Marseille hospital were associated with spring (April-June) (*p*<0.0001). No seasonal variation was associated with strains from Nice hospital.

### Multilocus Sequence Typing

**Figure 2:**
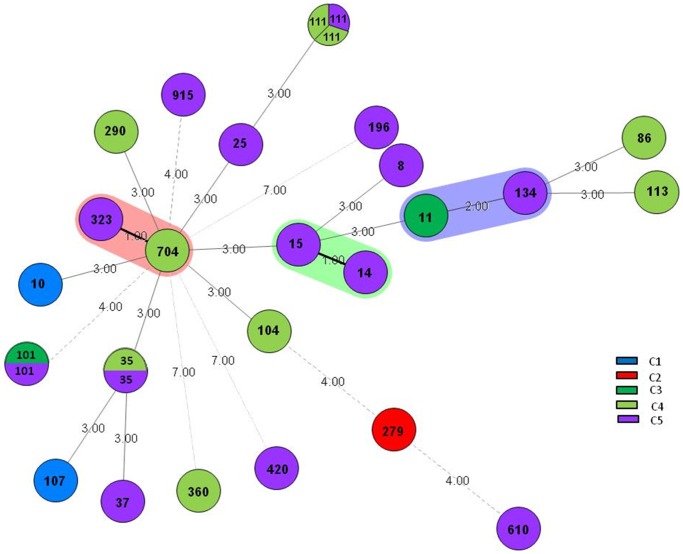
Mean spectra projection (MSP) dendrogram of *Klebsiella pneumoniae* strains generated by BIOTYPER software (version 2; Bruker Daltonics). ESBL: Extended-spectrum beta-lactamase, WT: wild type.

The MLST allelic profile of all strains distributed in the five clusters is presented in Table 4. From the 28 tested strains, we found 24 different sequence types (ST) (Figure 2). No relationship was observed between ST and the clinical and epidemiological data.

**Table 4:** Data mining analysis of *Klebsiella pneumoniae* MSP dendrogram.

Clusters (No)		Geographic location(No)		Type of samples (No)		Antibiotic resistancePhenotype (No)		Association between the antibioticresistance phenotype and typeof samples(No)		Seasonality(No)	
**C1+ C2**	23	Marseille	22	Urine	14	WT	19	Urine and WT	13	Spring	22
**C3**	65	Nice	47	Blood culturePus	2225	WT	35	Blood culture and WTUrine and WT	1718	SpringSummerAutumnWinter	1017146
**C4**	215	Marseille	117	Urine	74	WT	79	Urine and WT	56	Spring	62
		Angers	54	Urine	33	WT	34	Urine and WT	19	Winter	36
**C5**	232	Algeria	166	Tracheal aspirate	82	ESBL	149	Tracheal aspirate and ESBL	73	Winter	70

WT: wild type phenotype, ESBL: Extended Spectrum Beta-lactamase**.** No represents the number of strains that correspond to the significant character according to data mining analysis.

## Discussion

*K. pneumoniae* is an important pathogen with a complex pan-genome responsible for serious nosocomial infections, especially in intensive care units (ICUs) and in wards for surgery, emergency, neurology, pediatrics, and neonatology [5]. More concerning has been the emergence and increase in the isolation rate of ESBL-producing *K. pneumoniae* worldwide [4], which frequently possess resistance factors to other classes of antibiotics, notably aminoglycosides, fluoroquinolones and trimethoprim/sulfamethoxazole [21]. In our study, we confirmed that there is an increase in the number of multidrug-resistant *K. pneumoniae* strains with a considerable variation between the two countries studied. The antibiotic resistance rate in France (26.8%) was lower than that in Algeria (88.6%). In comparing our results with those of the Mystic study (1997–2003) [7] and another surveillance trial study (2004–2009) [22], we notice a worldwide north-south gradient evolution of ESBL production rate in *K. pneumoniae* strains with 12.3–12.8% in North America, 16.7% in Northern Europe, 24.4% in Southern Europe, 33.8% in the Middle East, 28.2–35.6% in Asia-Pacific, 45.5–51.9% in South America and 54.9% in Africa.

The high infection occurrence of *K. pneumoniae*, both ESBL-producing and non-producing strains, in Algerian and French hospitals pushed us to examine the epidemiology of these strains, which are considered to have a complex pan-genome containing plastic genome repertoires that differentiate strains according to their geographical locations, pathotypes, ecotypes and resistance phenotypes [23]. MALDI-TOF MS was successfully used as a tool for biotyping because we found specific clusters that were significantly associated with particular phenotypes from different clinical and geographical sources and from different seasons. This result is seemingly supported by Trevino et al., with a series of only 13 clinical isolates of *K. pneumoniae* [24]. By increasing the number of strains collected, our dendrogram became more refined, not only by country but also by hospital. This result could be of clinical importance for unknown pathogenic isolates, whose geographical sources could be detected rapidly using MALDI-TOF MS.

It is recognized that the distribution of bacteria may be related to geographical patterns, such as climatic zones and movement of human populations, and can provide information about pathogen evolution and transmission [25], [26]. The spatial distribution of bacterial pathogens can be considered at different levels; in a hospital setting, this may indicate nosocomial transmission, but in a community setting, the simultaneous appearance of an identical phenotype in widely dispersed locations may be a warning of an outbreak [15]. Our data also suggested that rates of *K. pneumoniae* infections varied seasonally and were significantly associated with periods of isolation that were due to changes in temperature and humidity. Several characteristics of this species that were previously described support our findings, which show that temperature and dew point were both linearly predictive of increasing rates of *K. pneumoniae* clinical isolates [27], [28]. Furthermore, most of the MALDI-TOF MS spectra are composed of well-conserved proteins with housekeeping functions that are minimally affected by environmental conditions and thus are considered to be optimal for the proteomic typing of bacteria, which is considerably different from genetic typing [29], [30].

By comparing the clusters obtained from the MSP dendrogram (Figure 1) with the ST distribution in the minimal spanning tree (MST) of *K. pneumoniae* strains (Figure 2), we noted that no correlation was observed for these two types of analysis. Moreover, no relationship was observed between ST, clinical and epidemiological data. These results suggest that the two approaches, comprising the MSP and MLST analyses, highlight two different bacterial aspects. Indeed, MLST analysis based on the conserved bacterial genes (the house-keeping genes) classifies bacteria according to their core genome, which represents less than 10% of the genome [31], while 90% of the genome is composed of “accessory genes” (mobile genetic elements) that can be lost or acquired by lateral gene transfer (LGT), and that is mainly responsible for the bacterial phenotype [23], [32]. Therefore, this core genome does not represent the majority of expressed proteins, in contrast to the MSP dendrogram analysis, which is based on the functional and expressed proteins of whole cells that is more representative of the global phenotype [33]. Therefore, these two approaches could be complementary to the extent that the MST approach, based on the analysis of the core genome, allows us to classify bacteria according to their conserved genes independent of their “accessory genes,” which can affect phenotypes and bacterial classification.

Many researchers have used molecular methods, particularly PFGE and MLST, to distinguish *K. pneumoniae* clinical isolates in order to understand transmission patterns and to aid in the management of these infections [34]. In 2008, a comparative study between these two methods found that PFGE is appropriate to discriminate among epidemiologically unrelated strains and appears more suitable for short-term epidemiology, while MLST is appropriate for strain phylogeny and large-scale epidemiology [35]. Furthermore, these molecular methods are costly and time-consuming to obtain results and introduce delays when attempting to limit the spread of *K. pneumoniae* clones [36]. For instance, only a few hours are required to obtain results by MALDI-TOF MS, whereas several days are necessary to collect MLST data [37]. In addition, the cost of MALDI-TOF MS instrumentation is comparable to that of a sequencing machine, but running costs and consumables are considerably lower than for these methods [38]. Barbuddhe et al. have used MALDI-TOF MS to accurately identify different *Listeria* species and correctly classified all *L. monocytogenes* serotypes in agreement with PFGE [38], and recently Wang et al. identified and classified *Streptococcus pyogenes* into clusters with MALDI-TOF MS [20].

Thus, MALDI-TOF MS combined with a statistical classification strategy is appropriate for studies of local epidemiology and global population structure when compared to a local database from clinical strains. It is a powerful epidemiological method and is sufficiently reproducible and sensitive enough to rapidly survey the evolution of existing or emerging phenotypes with reduced financial and human costs [39].

### Conclusion

We believe that our preliminary results should be expanded and confirmed with more strains obtained from different countries. To the best of our knowledge, this is the first study that analyzes the epidemiology of a large series of *K. pneumoniae* clinical isolates using MALDI-TOF MS application. We suggest the creation of a local database that is updated regularly to survey for the presence of abnormal phenotypes at the strain level. If a particular phenotype is detected, a real time genome sequencing approach could then be performed to investigate the origin and specific features of the strain. We believe that this methodology could be used routinely in clinical microbiology laboratories as a surveillance tool for hospital epidemiology studies to prevent outbreaks and dissemination of pathogens in hospital settings.

## Materials and Methods

### Bacterial Strains

**Figure 3:**
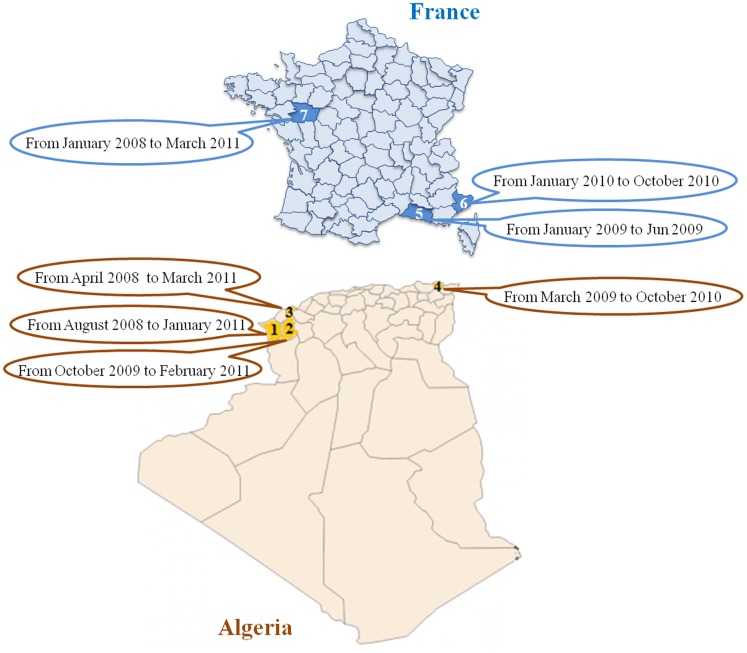
Minimal spanning tree (MST) of *K. pneumoniae* isolates, showing relationships between STs, compared with clusters obtained from the dendrogram generated by BIOTYPER software.

A total of 535 non redundant clinical strains of *K. pneumoniae*, isolated from different clinical samples, were collected during a period of 39 weeks, between January 2008 and March 2011. All of these were collected from hospitals in France and Algeria (Figure 3): Marseille hospitals, France (n = 170 strains); Angers hospital, France (n = 100 strains); Nice hospital, France (n = 54 strains), Tlemcen hospital, Algeria (n = 73 strains); Oran hospital, Algeria (n = 92 strains); Sidi Bel Abbes hospital, Algeria (n = 28 strains) and Annaba hospital, Algeria (n = 18 strains). The clinical sources of the different strains are noted in Table 5.

**Table 5:** MLST allelic profile of *Klebsiella pneumoniae* clinical isolates distributed in the five clusters.

Clusters	Isolates	Hospitals	Sample origin	Antibiotic resistance phenotype	gapA	infB	mdh	pgi	phoE	rpoB	tonB	ST
**C1**	KpM 44	Marseille	Urine	WT	2	1	2	17	27	1	39	**ST107**
	KpM 71	Marseille	Pus	WT	4	1	7	1	9	1	12	**ST10**
**C2**	KpnA1211	Annaba	Tracheal aspirate	ESBL	3	3	1	1	1	1	4	**ST11**
	KpM 29	Marseille	Blood culture	ESBL	2	6	1	5	4	1	6	**ST101**
**C3**	29 Kp	Nice	Pus	WT	2	2	1	47	1	4	43	**ST279**
	KpM 154	Marseille	Urine	WT	2	1	5	1	17	4	42	**ST111**
	KpnA 1323	Annaba	Tracheal aspirate	ESBL	2	1	1	1	9	1	20	**ST704**
	16 KP	Nice	Pus	WT	2	1	5	1	17	4	42	**ST111**
	16 KH	Nice	Blood culture	WT	14	1	2	1	21	1	23	**ST113**
	KpA4	Angers	Vaginal swab	WT	16	24	21	53	47	17	67	**ST360**
	KpA63	Angers	Tracheal aspirate	WT	9	4	2	1	1	1	27	**ST86**
	Kpn8	Oran	Rectal swab	Pase High Level	2	1	1	37	10	1	86	**ST290**
	14 KP	Nice	Pus	WT	4	5	1	29	1	4	42	**ST610**
	10 KP	Nice	Pus	ESBL	2	1	5	1	17	4	42	**ST111**
**C4**	Kpn9	Oran	Tracheal aspirate	WT	2	3	1	1	2	1	43	**ST104**
	KpM 166	Marseille	ND	ESBL	1	1	1	1	1	1	1	**ST15**
	KpM 161	Marseille	ND	ESBL	17	19	39	39	51	72	72	**ST196**
	KpA86	Angers	Urine	WT	3	1	2	1	1	1	4	**ST134**
	KpA75	Angers	Blood culture	WT	2	6	1	5	4	1	6	**ST101**
	Okp46	Oran	Tracheal aspirate	ESBL+Case	2	1	2	1	10	1	19	**ST35**
	Okp45	Oran	Tracheal aspirate	ESBL	1	6	1	1	1	1	1	**ST14**
	Kp98	Tlemcen	Rectal swab	ESBL	2	1	1	1	10	4	13	**ST25**
**C5**	KpM 170	Marseille	Blood culture	ESBL	2	1	2	1	10	1	19	**ST35**
	Kp90	Tlemcen	Pus	ESBL	2	10	13	1	12	1	186	**ST915**
	Skp25	Sidi Bel Abbes	Pus	ESBL	2	1	1	1	9	1	93	**ST323**
	Skp22	Sidi Bel Abbes	Bedsore	ESBL	2	1	65	2	5	1	36	**ST420**
	KpnA 932	Annaba	Tracheal aspirate	ESBL	2	9	2	1	13	1	16	**ST37**
	KpnA 576	Annaba	Urine	ESBL	4	1	1	1	1	5	6	**ST8**

ESBL: Extended-spectrum beta-lactamase, ESBL+Case: Extended-spectrum beta-lactamase associated to Cephalosporinase phenotype, Pase: Penicillinase phenotype, WT: Wild Type phenotype.

### *Klebsiella pneumoniae* Identification using MALDI-TOF MS

Isolates were plated on Trypticase Soy Agar (BioMerieux) and incubated for 24 h at 37°C. One single colony from each isolate was deposited on a MALDI-TOF MTP 384 target plate (Bruker Daltonics, Bremen, Germany) in four replicates to minimize random effects. Two microliters of matrix solution (saturated α-cyano-4-hydroxycinnamic acid, 50% acetonitrile, 2.5% trifluoroacetic acid) were then added and allowed to co-crystallize with the sample. Analysis was performed in a MALDI-TOF MS spectrometer (337 nm) (Autoflex; Bruker Daltonics) with FLEX control software (Bruker Daltonics). Ions were accelerated in the positive ion mode with an accelerating voltage of 20 kV. The pulsed extraction of ions was optimized for 1000 Da. The software employed, Bruker Biotyper 2.0 (Bruker Daltonics), automatically acquired spectra with fuzzy control of the laser intensity and analyzed them by standard pattern matching against the spectra of 2881 species used as reference data. After comparing the unknown spectra with all reference spectra in the database, the log scores were ranked. Values of >1.9 were required for secure identification at the species level, and values between 1.9 and 1.7 were required for secure identification at the genus level.

### Clustering of MALDI-TOF Spectra

A consensus spectrum was produced, and an MSP dendrogram was constructed using the correlation distance measure with the average linkage algorithm setting of the Biotyper 2.0 software. Clusters were then detailed and analyzed according to arbitrary distance levels at 500, 180 and 100.

### Antibiotic Susceptibility and Synergy Testing

Antibiotic susceptibility testing was performed using the disk diffusion method on Mueller-Hinton medium as per the guidelines of the French Society of Microbiology (www.sfm.asso.fr). *E. coli* ATCC 25922 was used as a quality control strain. The antimicrobial disks tested were as follows: ampicillin (10 µg), amoxicillin (25 µg), amoxicillin/clavulanic acid (20/10 µg), ticarcillin (75 µg), cefalotin (30 µg), cefoxitin (30 µg), ceftazidime (30 µg), cefotaxime (30 µg), ceftriaxone (30 µg), imipenem (10 µg), gentamicin (15 µg), amikacin (30 µg), tobramycin (10 µg), ciprofloxacin (5 µg), trimethoprim/sulfamethoxazole (1,25/23,75 µg), and colistin (50 µg). ESBL production was detected using the method of combined antibiotic disks as previously described [40].

### Antibiotic Resistance Phenotypic Classification of *K. pneumoniae* Strains based on Beta-Lactamine Compounds

We considered a wild type phenotype as a strain that confer resistance to aminopenicillins, carboxypenicillins and to ureidopenicillins. The high level penicillinase phenotype was presented by a high penicillinase activity responsible for resistance to aminopenicillins and their inhibitors, to carboxypenicillins, to ureidopenicillins, and to first generation cephalosporins (1 GC). The inhibitor-resistant TEM penicillinase phenotype included resistance to aminopenicillins, carboxypenicillins, and ureidopenicillins. It was distinguished by resistance to aminopenicillins and carbocxypenicillins associated to the beta-lactam inhibitors. 1 GC generally retain their activity. The cephalosporinase phenotype corresponded to a marked resistance to penicillins, 1 GC, 2 GC, and to at least one 3 GC. The extended spectrum B-lactamase phenotype includes resistance to penicillins and cephalosporins except cephamycins. The resistance to 3 GC and 4 GC was more or less pronounced depending on the enzymes and the strains.

### Statistical Analysis

Clinical and epidemiological data were recorded in an Excel file (Microsoft, Redmond, WA, USA), including the clustering obtained using the MSP dendrogram generated by the Biotyper software (version 2; Bruker Daltonics), and were analyzed using PASW Statistics software version 17.0 (SPSS Inc., Chicago, IL, USA). Dependent variable series were analyzed using Expert Modeler, which automatically generates the best-fitting model. The chi-square analysis was used also to compare proportions using the same software, and *P* values <0.05 were considered to be statistically significant. Statistical analyses were conducted using Epi Info version 6 (Centers for Disease Control and Prevention, Atlanta, GA, USA).

### Multilocus Sequence Typing

Randomly, 28 strains distributed in the five clusters were selected to perform the full MLST as described previously using seven housekeeping genes, including *gapA*, *infB*, *mdh*, *pgi*, *phoE*, *rpoB* and *tonB* [34]. The MLST database for *K. pneumoniae* can be found at http://www.pasteur.fr. Based on the allelic profiles, the evolutionary relationship between isolates was assessed by the minimal spanning tree (MST) algorithm in Bionumerics (Applied Maths, NV St-Martens-Latem, Belgium). A stringent definition of 6/7 shared alleles was used to define clonal complexes (single locus variants only).

## Supporting Information

Table S1 Distribution of *Klebsiella pneumoniae* strains according to the dendrogram generated by BIOTYPER software (version 2, Bruker Daltonics) at the distance level of 500.
(DOCX)

Table S2 Comparison of the clusters obtained according to different cut-offs at 500, 180 and 100 of the dendrogram generated by BIOTYPER software (version 2, Bruker Daltonics).
(DOCX)
