# Supplementary material for: Biotyping of Multidrug-Resistant Klebsiella pneumoniae Clinical Isolates from France and Algeria Using MALDI-TOF MS
Source: PLoS One. 2013 Apr 19;8(4):e61428. doi: 10.1371/journal.pone.0061428 (PMC3631213; doi:10.1371/journal.pone.0061428)
Supplement: Table S1 — Distribution of Klebsiella pneumoniae strains according to the dendrogram generated by BIOTYPER software (version 2, Bruker Daltonics) at the distance level of 500. (DOCX) [file pone.0061428.s001.docx]

**Table S1.** Distribution of *Klebsiella pneumoniae* strains according to the dendrogram generated by BIOTYPER software (version 2, Bruker Daltonics) at the distance level of 500.

| **Clusters (Number)** | | **Cities (Number)** | | **Type of samples (Number)** | | **Phenotype (Number)** | |
| --- | --- | --- | --- | --- | --- | --- | --- |
| **C1** | **16** | Marseille | 16 | Urine | 9 | WT | 8 |
|  |  |  |  |  |  | ESBL | 1 |
|  |  |  |  | Blood culture | 2 | ESBL | 1 |
|  |  |  |  |  |  | WT | 1 |
|  |  |  |  | Bedsore | 1 | WT | 1 |
|  |  |  |  | Profound swab | 2 | WT | 2 |
|  |  |  |  | Pus | 2 | WT | 2 |
| **C2** | **7** | Annaba | 1 | Tracheal aspirate | 1 | ESBL | 1 |
|  |  | Marseille | 6 | Blood culture | 1 | ESBL | 1 |
|  |  |  |  | Urine | 5 | WT | 5 |
| **C3** | **65** | Annaba | 1 | Tracheal aspirate | 1 | ESBL | 1 |
|  |  | Angers | 2 | Tracheal aspirate | 1 | WT | 1 |
|  |  |  |  | Vaginal swab | 1 | WT | 1 |
|  |  | Marseille | 10 | Urine | 8 | WT | 7 |
|  |  |  |  |  |  | ESBL | 1 |
|  |  |  |  | Blood culture | 1 | ESBL | 1 |
|  |  |  |  | Not determined | 1 | ESBL | 1 |
|  |  | Oran | 5 | Rectal swab | 1 | Pase HL | 1 |
|  |  |  |  | Tracheal aspirate | 1 | ESBL | 1 |
|  |  |  |  | Pus | 1 | ESBL | 1 |
|  |  |  |  | Urine | 1 | WT | 1 |
|  |  |  |  | Environment | 1 | ESBL | 1 |
|  |  | Nice | 47 | Blood culture | 22 | ESBL | 2 |
|  |  |  |  |  |  | Pase HL | 3 |
|  |  |  |  |  |  | WT | 17 |
|  |  |  |  | Pus | 25 | ESBL | 3 |
|  |  |  |  |  |  | Pase HL | 3 |
|  |  |  |  |  |  | WT | 18 |
|  |  |  |  |  |  | Pase IRT | 1 |
| **C4** | **215** | Annaba | 1 | Tracheal aspirate | 1 | ESBL | 1 |
|  |  | Angers | 54 | Catheter | 2 | ESBL + Case | 1 |
|  |  |  |  |  |  | WT | 1 |
|  |  |  |  | Tracheal aspirate | 2 | WT | 2 |
|  |  |  |  | Urine | 33 | WT | 19 |
|  |  |  |  |  |  | Pase IRT | 1 |
|  |  |  |  |  |  | Case | 2 |
|  |  |  |  |  |  | ESBL | 9 |
|  |  |  |  |  |  | ESBL + Case | 2 |
|  |  |  |  | Blood culture | 12 | ESBL | 3 |
|  |  |  |  |  |  | WT | 9 |
|  |  |  |  | Rectal swab | 4 | WT | 2 |
|  |  |  |  |  |  | ESBL + Case | 2 |
|  |  |  |  | Pus | 1 | WT | 1 |
|  |  | Nice | 6 | Blood culture | 3 | WT | 1 |
|  |  |  |  |  |  | Pase HL | 1 |
|  |  |  |  |  |  | ESBL | 1 |
|  |  |  |  | Pus | 3 | ESBL | 1 |
|  |  |  |  |  |  | WT | 2 |
|  |  | Oran | 14 | Pus | 2 | WT | 1 |
|  |  |  |  |  |  | ESBL | 1 |
|  |  |  |  | Rectal swab | 2 | ESBL | 2 |
|  |  |  |  | Tracheal aspirate | 8 | WT | 3 |
|  |  |  |  |  |  | ESBL | 1 |
|  |  |  |  |  |  | ESBL + Case | 4 |
|  |  |  |  | Environment | 2 | ESBL | 1 |
|  |  |  |  |  |  | ESBL + Case | 1 |
|  |  | Sidi  Bel Abbes | 10 | Tracheal aspirate | 2 | ESBL | 1 |
|  |  |  |  |  |  | ESBL + Case | 1 |
|  |  |  |  | Urine | 3 | ESBL | 2 |
|  |  |  |  |  |  | Pase IRT | 1 |
|  |  |  |  | Environment | 1 | ESBL | 1 |
|  |  |  |  | Pus | 4 | ESBL | 3 |
|  |  |  |  |  |  | ESBL + Case | 1 |
|  |  | Tlemcen | 13 | Tracheal aspirate | 5 | ESBL | 5 |
|  |  |  |  | Urine | 1 | Pase HL | 1 |
|  |  |  |  | Environment | 1 | ESBL | 1 |
|  |  |  |  | Pus | 1 | ESBL | 1 |
|  |  |  |  | Rectal swab | 5 | ESBL | 3 |
|  |  |  |  |  |  | ESBL + Case | 2 |
|  |  | Marseille | 117 | Tracheal aspirate | 8 | ESBL | 3 |
|  |  |  |  |  |  | WT | 5 |
|  |  |  |  | Urine | 74 | WT | 56 |
|  |  |  |  |  |  | Pase IRT | 2 |
|  |  |  |  |  |  | ESBL | 16 |
|  |  |  |  | Blood culture | 14 | ESBL | 3 |
|  |  |  |  |  |  | WT | 11 |
|  |  |  |  | Profound swab | 4 | WT | 3 |
|  |  |  |  |  |  | ESBL | 1 |
|  |  |  |  | Pus | 3 | ESBL | 1 |
|  |  |  |  |  |  | WT | 2 |
|  |  |  |  | Not determined | 13 | WT | 2 |
|  |  |  |  |  |  | ESBL | 11 |
|  |  |  |  | Catheter | 1 | ESBL | 1 |
| **C5** | **232** | Oran | 74 | Tracheal aspirate | 43 | ESBL | 31 |
|  |  |  |  |  |  | ESBL + Case | 7 |
|  |  |  |  |  |  | WT | 5 |
|  |  |  |  | Urine | 12 | WT | 3 |
|  |  |  |  |  |  | ESBL + Case | 1 |
|  |  |  |  |  |  | ESBL | 8 |
|  |  |  |  | Pus | 10 | ESBL + Case | 1 |
|  |  |  |  |  |  | ESBL | 9 |
|  |  |  |  | Rectal swab | 6 | ESBL | 3 |
|  |  |  |  |  |  | ESBL + Case | 2 |
|  |  |  |  |  |  | Pase HL | 1 |
|  |  |  |  | Vaginal swab | 1 | ESBL | 1 |
|  |  |  |  | Environment | 2 | ESBL | 2 |
|  |  | Angers | 44 | Catheter | 1 | ESBL | 1 |
|  |  |  |  | Urine | 25 | ESBL | 4 |
|  |  |  |  |  |  | ESBL + Case | 1 |
|  |  |  |  |  |  | Pase IRT | 1 |
|  |  |  |  |  |  | WT | 19 |
|  |  |  |  | Blood culture | 6 | WT | 5 |
|  |  |  |  |  |  | ESBL | 1 |
|  |  |  |  | Rectal swab | 5 | ESBL | 5 |
|  |  |  |  | Vaginal swab | 5 | ESBL | 1 |
|  |  |  |  |  |  | WT | 4 |
|  |  |  |  | Pus | 2 | WT | 2 |
|  |  | Tlemcen | 59 | Tracheal aspirate | 23 | WT | 3 |
|  |  |  |  |  |  | ESBL | 19 |
|  |  |  |  |  |  | ESBL + Case | 1 |
|  |  |  |  | Profound swab | 1 | ESBL | 1 |
|  |  |  |  | Rectal swab | 13 | ESBL | 12 |
|  |  |  |  |  |  | ESBL + Case | 1 |
|  |  |  |  | Pus | 10 | ESBL + Case | 2 |
|  |  |  |  |  |  | ESBL | 8 |
|  |  |  |  | Environment | 10 | ESBL | 6 |
|  |  |  |  |  |  | Case | 1 |
|  |  |  |  |  |  | ESBL + Case | 3 |
|  |  |  |  | Urine | 2 | ESBL | 2 |
|  |  | Marseille | 21 | Pus | 1 | ESBL | 1 |
|  |  |  |  | Subcutaneous swab | 1 | WT | 1 |
|  |  |  |  | Not determined | 3 | ESBL | 3 |
|  |  |  |  | Blood culture | 3 | WT | 3 |
|  |  |  |  | Urine | 12 | WT | 7 |
|  |  |  |  |  |  | ESBL | 5 |
|  |  |  |  | Tracheal aspirate | 1 | WT | 1 |
|  |  | Annaba | 15 | Tracheal aspirate | 9 | WT | 1 |
|  |  |  |  |  |  | ESBL | 8 |
|  |  |  |  | Urine | 4 | ESBL | 4 |
|  |  |  |  | Blood culture | 2 | ESBL | 2 |
|  |  | Sidi  Bel Abbes | 18 | Pus | 7 | ESBL | 4 |
|  |  |  |  |  |  | ESBL + Case | 1 |
|  |  |  |  |  |  | Pase IRT | 1 |
|  |  |  |  |  |  | WT | 1 |
|  |  |  |  | Profound swab | 1 | Pase HL | 1 |
|  |  |  |  | Bedsore | 2 | ESBL | 2 |
|  |  |  |  | Tracheal aspirate | 7 | ESBL | 7 |
|  |  |  |  | Environment | 1 | ESBL | 1 |
|  |  | Nice | 1 | Blood culture | 1 | WT | 1 |

ESBL: Extended-spectrum beta-lactamase, Case: Cephalosporinase phenotype, ESBL + Case: Extended-spectrum beta-lactamase associated to Cephalosporinase phenotype, Pase HL: Penicillinase High Level phenotype, Pase IRT: inhibitor-resistant TEM penicillinase, WT: Wild Type phenotype.
