# Supplementary material for: Biotyping of Multidrug-Resistant Klebsiella pneumoniae Clinical Isolates from France and Algeria Using MALDI-TOF MS
Source: PLoS One. 2013 Apr 19;8(4):e61428. doi: 10.1371/journal.pone.0061428 (PMC3631213; doi:10.1371/journal.pone.0061428)
Supplement: Table S2 — Comparison of the clusters obtained according to different cut-offs at 500, 180 and 100 of the dendrogram generated by BIOTYPER software (version 2, Bruker Daltonics). (DOCX) [file pone.0061428.s002.docx]

**Table S2.** Comparison of the clusters obtained according to different cut-offs at 500, 180 and 100 of the dendrogram generated by BIOTYPER software (version 2, Bruker Daltonics).

| **Cut-off at 500** | | | | **Cut-off at 180** | | | | | **Cut-off at 100** | | | | |
| --- | --- | --- | --- | --- | --- | --- | --- | --- | --- | --- | --- | --- | --- |
| **Clusters (Num)** | | **Cities (Num)** | | **Num of clusters** | **Clusters (Num)** | | **Cities (Num)** | | **Num of clusters** | **Clusters (Num)** | | **Cities (Num)** | |
| **C1** | **16** | Marseille | 16 | 2 clusters | C1 | 3 | Marseille | 3 | 1 cluster | C1 | 8 | Marseille | 8 |
|  |  |  |  |  | C2 | 10 | Marseille | 10 |  |  |  |  |  |
| **C2** | **7** | Annaba  Marseille | 1  6 | 1 cluster | C1 | 2 | Marseille | 2 | No clusters | | | | |
| **C3** | **65** | Annaba  Angers  Marseille  Oran  Nice | 1  2  10  5  47 | 8 clusters | C1 | 2 | Nice | 2 | 8 clusters | C1 | 3 | Nice | 3 |
|  |  |  |  |  | C2 | 16 | Nice | 16 |  | C2 | 2 | Nice | 2 |
|  |  |  |  |  | C3 | 2 | Marseille | 1 |  | C3 | 2 | Nice | 2 |
|  |  |  |  |  |  |  | Nice | 1 |  |  |  |  |  |
|  |  |  |  |  | C4 | 8 | Nice | 8 |  | C4 | 1 | Nice | 2 |
|  |  |  |  |  | C5 | 7 | Marseille | 4 |  | C5 | 3 | Nice | 1 |
|  |  |  |  |  |  |  | Nice | 3 |  |  |  | Marseille | 2 |
|  |  |  |  |  | C6 | 7 | Oran | 3 |  | C6 | 2 | Nice | 1 |
|  |  |  |  |  |  |  | Nice | 4 |  |  |  | Oran | 1 |
|  |  |  |  |  | C7 | 3 | Nice | 3 |  | C7 | 2 | Nice | 2 |
|  |  |  |  |  | C8 | 7 | Nice | 7 |  | C8 | 2 | Nice | 2 |
| **C4** | **215** | Annaba  Angers  Nice  Oran  Sidi Bel Abbes  Tlemcen  Marseille | 1  54  6  14  10  13  117 | 16 clusters | C1 | 3 | Marseille | 3 | 26 clusters | C1 | 2 | Marseille | 2 |
|  |  |  |  |  | C2 | 4 | Marseille | 4 |  | C2 | 2 | Marseille | 2 |
|  |  |  |  |  | C3 | 4 | Marseille | 4 |  | C3 | 3 | Marseille | 3 |
|  |  |  |  |  | C4 | 3 | Marseille | 3 |  | C4 | 2 | Marseille | 2 |
|  |  |  |  |  | C5 | 10 | Marseille | 10 |  | C5 | 2 | Marseille | 2 |
|  |  |  |  |  | C6 | 2 | Marseille | 2 |  | C6 | 3 | Marseille | 3 |
|  |  |  |  |  | C7 | 43 | Marseille | 43 |  | C7 | 3 | Marseille | 3 |
|  |  |  |  |  | C8 | 4 | Marseille | 4 |  | C8 | 3 | Marseille | 3 |
|  |  |  |  |  | C9 | 4 | Marseille | 4 |  | C9 | 15 | Marseille | 15 |
|  |  |  |  |  | C10 | 14 | Marseille | 14 |  | C10 | 7 | Marseille | 7 |
|  |  |  |  |  | C11 | 17 | Marseille | 17 |  | C11 | 14 | Marseille | 14 |
|  |  |  |  |  | C12 | 4 | Marseille | 1 |  | C12 | 3 | Marseille | 3 |
|  |  |  |  |  |  |  | Oran | 1 |  |  |  |  |  |
|  |  |  |  |  |  |  | Angers | 1 |  |  |  |  |  |
|  |  |  |  |  |  |  | Nice | 1 |  |  |  |  |  |
|  |  |  |  |  | C13 | 27 | Angers | 15 |  | C13 | 2 | Marseille | 2 |
|  |  |  |  |  |  |  | Oran | 6 |  |  |  |  |  |
|  |  |  |  |  |  |  | Sidi Bel Abbes | 1 |  |  |  |  |  |
|  |  |  |  |  |  |  | Tlemcen | 1 |  |  |  |  |  |
|  |  |  |  |  |  |  | Marseille | 2 |  |  |  |  |  |
|  |  |  |  |  |  |  | Nice | 2 |  |  |  |  |  |
|  |  |  |  |  | C14 | 2 | Sidi Bel Abbes | 1 |  | C14 | 2 | Marseille | 2 |
|  |  |  |  |  |  |  | Nice | 1 |  |  |  |  |  |
|  |  |  |  |  | C15 | 6 | Angers | 6 |  | C15 | 3 | Marseille | 3 |
|  |  |  |  |  | C16 | 63 | Angers | 32 |  | C16 | 4 | Marseille | 4 |
|  |  |  |  |  |  |  | Oran | 7 |  |  |  |  |  |
|  |  |  |  |  |  |  | Marseille | 1 |  |  |  |  |  |
|  |  |  |  |  |  |  | Sidi Bel Abbes | 8 |  |  |  |  |  |
|  |  |  |  |  |  |  | Tlemcen | 12 |  |  |  |  |  |
|  |  |  |  |  |  |  | Nice | 2 |  |  |  |  |  |
|  |  |  |  |  |  |  | Annaba | 1 |  |  |  |  |  |
|  |  |  |  |  |  |  |  |  |  | C17 | 7 | Marseille | 7 |
|  |  |  |  |  |  |  |  |  |  | C18 | 9 | Marseille | 9 |
|  |  |  |  |  |  |  |  |  |  | C19 | 22 | Angers | 13 |
|  |  |  |  |  |  |  |  |  |  |  |  | Oran | 6 |
|  |  |  |  |  |  |  |  |  |  |  |  | Sidi Bel Abbes | 1 |
|  |  |  |  |  |  |  |  |  |  |  |  | Tlemcen | 1 |
|  |  |  |  |  |  |  |  |  |  |  |  | Marseille | 1 |
|  |  |  |  |  |  |  |  |  |  | C20 | 3 | Nice | 1 |
|  |  |  |  |  |  |  |  |  |  |  |  | Angers | 1 |
|  |  |  |  |  |  |  |  |  |  |  |  | Marseille | 1 |
|  |  |  |  |  |  |  |  |  |  | C21 | 4 | Angers | 4 |
|  |  |  |  |  |  |  |  |  |  | C22 | 10 | Angers | 8 |
|  |  |  |  |  |  |  |  |  |  |  |  | Oran | 1 |
|  |  |  |  |  |  |  |  |  |  |  |  | Marseille | 1 |
|  |  |  |  |  |  |  |  |  |  | C23 | 3 | Tlemcen | 2 |
|  |  |  |  |  |  |  |  |  |  |  |  | Sidi Bel Abbes | 1 |
|  |  |  |  |  |  |  |  |  |  | C24 | 19 | Angers | 9 |
|  |  |  |  |  |  |  |  |  |  |  |  | Sidi Bel Abbes | 4 |
|  |  |  |  |  |  |  |  |  |  |  |  | Tlemcen | 5 |
|  |  |  |  |  |  |  |  |  |  |  |  | Oran | 1 |
|  |  |  |  |  |  |  |  |  |  | C25 | 28 | Angers | 15 |
|  |  |  |  |  |  |  |  |  |  |  |  | Oran | 4 |
|  |  |  |  |  |  |  |  |  |  |  |  | Marseille | 1 |
|  |  |  |  |  |  |  |  |  |  |  |  | Sidi Bel Abbes | 2 |
|  |  |  |  |  |  |  |  |  |  |  |  | Tlemcen | 5 |
|  |  |  |  |  |  |  |  |  |  |  |  | Nice | 1 |
|  |  |  |  |  |  |  |  |  |  | C26 | 3 | Angers | 1 |
|  |  |  |  |  |  |  |  |  |  |  |  | Oran | 1 |
|  |  |  |  |  |  |  |  |  |  |  |  | Sidi Bel Abbes | 1 |
| **C5** | **232** | Oran  Angers  Tlemcen  Marseille  Annaba  Sidi Bel Abbes  Nice | 74  44  59  21  15  18  1 | 7 clusters | C1 | 3 | Annaba | 2 | 17 Clusters | C1 | 2 | Annaba | 2 |
|  |  |  |  |  |  |  | Marseille | 1 |  |  |  |  |  |
|  |  |  |  |  | C2 | 19 | Marseille | 19 |  | C2 | 5 | Marseille | 5 |
|  |  |  |  |  | C3 | 3 | Annaba | 3 |  | C3 | 4 | Marseille | 4 |
|  |  |  |  |  | C4 | 2 | Oran | 1 |  | C4 | 7 | Marseille | 7 |
|  |  |  |  |  |  |  | Angers | 1 |  |  |  |  |  |
|  |  |  |  |  | C5 | 93 | Angers | 32 |  | C5 | 2 | Annaba | 2 |
|  |  |  |  |  |  |  | Oran | 23 |  |  |  |  |  |
|  |  |  |  |  |  |  | Annaba | 4 |  |  |  |  |  |
|  |  |  |  |  |  |  | Marseille | 1 |  |  |  |  |  |
|  |  |  |  |  |  |  | Sidi Bel Abbes | 13 |  |  |  |  |  |
|  |  |  |  |  |  |  | Tlemcen | 20 |  |  |  |  |  |
|  |  |  |  |  | C6 | 6 | Nice | 1 |  | C6 | 2 | Angers | 1 |
|  |  |  |  |  |  |  | Oran | 1 |  |  |  | Oran | 1 |
|  |  |  |  |  |  |  | Angers | 4 |  |  |  |  |  |
|  |  |  |  |  | C7 | 106 | Tlemcen | 39 |  | C7 | 2 | Annaba | 2 |
|  |  |  |  |  |  |  | Sidi Bel Abbes | 5 |  |  |  |  |  |
|  |  |  |  |  |  |  | Oran | 49 |  |  |  |  |  |
|  |  |  |  |  |  |  | Angers | 7 |  |  |  |  |  |
|  |  |  |  |  |  |  | Annaba | 5 |  |  |  |  |  |
|  |  |  |  |  |  |  | Marseille | 1 |  |  |  |  |  |
|  |  |  |  |  |  |  |  |  |  | C8 | 2 | Oran | 2 |
|  |  |  |  |  |  |  |  |  |  | C9 | 3 | Oran | 2 |
|  |  |  |  |  |  |  |  |  |  |  |  | Sidi Bel Abbes | 1 |
|  |  |  |  |  |  |  |  |  |  | C10 | 2 | Angers | 1 |
|  |  |  |  |  |  |  |  |  |  |  |  | Oran | 1 |
|  |  |  |  |  |  |  |  |  |  | C11 | 12 | Angers | 5 |
|  |  |  |  |  |  |  |  |  |  |  |  | Oran | 4 |
|  |  |  |  |  |  |  |  |  |  |  |  | Sidi Bel Abbes | 3 |
|  |  |  |  |  |  |  |  |  |  | C12 | 71 | Angers | 26 |
|  |  |  |  |  |  |  |  |  |  |  |  | Annaba | 2 |
|  |  |  |  |  |  |  |  |  |  |  |  | Oran | 13 |
|  |  |  |  |  |  |  |  |  |  |  |  | Marseille | 1 |
|  |  |  |  |  |  |  |  |  |  |  |  | Sidi Bel Abbes | 9 |
|  |  |  |  |  |  |  |  |  |  |  |  | Tlemcen | 20 |
|  |  |  |  |  |  |  |  |  |  | C13 | 3 | Angers | 3 |
|  |  |  |  |  |  |  |  |  |  | C14 | 51 | Angers | 4 |
|  |  |  |  |  |  |  |  |  |  |  |  | Annaba | 3 |
|  |  |  |  |  |  |  |  |  |  |  |  | Marseille | 1 |
|  |  |  |  |  |  |  |  |  |  |  |  | Oran | 24 |
|  |  |  |  |  |  |  |  |  |  |  |  | Sidi Bel Abbes | 1 |
|  |  |  |  |  |  |  |  |  |  |  |  | Tlemcen | 18 |
|  |  |  |  |  |  |  |  |  |  | C15 | 3 | Sidi Bel Abbes | 1 |
|  |  |  |  |  |  |  |  |  |  |  |  | Oran | 1 |
|  |  |  |  |  |  |  |  |  |  |  |  | Angers | 1 |
|  |  |  |  |  |  |  |  |  |  | C16 | 2 | Oran | 1 |
|  |  |  |  |  |  |  |  |  |  |  |  | Tlemcen | 1 |
|  |  |  |  |  |  |  |  |  |  | C17 | 45 | Angers | 2 |
|  |  |  |  |  |  |  |  |  |  |  |  | Annaba | 1 |
|  |  |  |  |  |  |  |  |  |  |  |  | Oran | 22 |
|  |  |  |  |  |  |  |  |  |  |  |  | Sidi Bel Abbes | 3 |
|  |  |  |  |  |  |  |  |  |  |  |  | Tlemcen | 17 |
